# Successful treatment using combined electroconvulsive therapy and oral paliperidone for clozapine-resistant schizophrenia: A case report

**DOI:** 10.1192/j.eurpsy.2023.2162

**Published:** 2023-07-19

**Authors:** C. I. Varlam, V. Dionisie, G. Andrișca, M. Manea

**Affiliations:** 1 4th Department, “Prof. Dr. Alexandru Obregia” Clinical Hospital of Psychiatry, Bucharest, Romania

## Abstract

**Introduction:**

Clozapine is considered to be the most efficacious antipsychotic drug for treatment-resistant schizophrenia (TRS). Despite this, up to 70% of patients with TRS have a poor response to adequate treatment with clozapine. In order to overcome clozapine-resistance schizophrenia (CRS), a number of adjunctive therapies, including pharmacological and non-pharmacological options, have been attempted.

**Objectives:**

The objective of this paper is to highlight the efficacy of the combined electroconvulsive therapy (ECT) and oral paliperidone as a successful treatment in clozapine nonresponders suffering from schizophrenia.

**Methods:**

We present the case of a 22-year-old female, with four years psychiatric history, which was admitted to our clinic for psychotic behavior, psychomotor agitation, verbal negativism, auditory hallucinations. During hospitalization, the patient presented behavioral disorganization, auditory, visual and tactile hallucinations, ideo-verbal barriers, poorly systematized delusional ideation (of guilt, mysticism, contamination, possession), episodes of catatonic stupor, rigidity, waxy flexibility, bizarre postures, false recognitions. Corroborating evidence, we established the diagnosis of undifferentiated schizophrenia. We initiated treatment with clozapine up to 450 mg/day and amisulpride up to 600 mg/day.

**Results:**

Combined treatment strategy of clozapine and amisulpride for six weeks showed no amelioration in our patient, with additional side-effects. Also, in the last four years, she had been treated with several atypical antipsychotics, which had not achieved substantial improvement. Considering that our patient did not present an adequate clinical response and the catatonic symptoms were accentuated, we decided to progressively reduce the doses of clozapine by 50 mg/day until elimination, to initiate paliperidone 12 mg/day and to conduct ECT three times a week, performing a total of six sessions. The bilateral electrode placement and brief pulse stimuli (800 mA; 8 s, 30 Hz) were applied under analgo-sedation, with no sustained severe adverse events. After performing ECT, the patient presented a favorable clinical evolution, with a decreasing trend until the remission of psychotic symptoms.

**Conclusions:**

TRS was diagnosed based on the poor response to more than two kinds of atypical antipsychotics and CRS was established after the combination of clozapine and amisulpride failed to decrease persistent positive symptoms, associated with worsening of the negative symptoms. Combined therapy with paliperidone and ECT proved to be greatly effective in improving symptoms for our patient. Switching from clozapine to a previously untried atypical might be of benefit in TRS. Also, adjunctive ECT can be efficacious in CRS. Augmentation with ECT may result in a faster response, which is particularly useful among patients with high risks of self-harm.

**Disclosure of Interest:**

None Declared

